# Influence of Contextual Variables in the Changes of Direction and Centripetal Force Generated during an Elite-Level Soccer Team Season

**DOI:** 10.3390/ijerph17030967

**Published:** 2020-02-04

**Authors:** Paulino Granero-Gil, Alejandro Bastida-Castillo, Daniel Rojas-Valverde, Carlos D. Gómez-Carmona, Ernesto de la Cruz Sánchez, José Pino-Ortega

**Affiliations:** 1 Department of Physical Activity and Sport, International Excellence Campus “Mare Nostrum”, Sport Science Faculty, University of Murcia, 30720 San Javier, Murcia, Spain; paulino.granero@um.es (P.G.-G.); erneslacruz@um.es (E.d.l.C.S.); josepinoortega@um.es (J.P.-O.); 2 Fitness Coach of PFC CSKA Moscow and Russian Football Union, 125252 Moscow, Russia; 3 Centre of Research and Diagnosis in Health and Sport (CIDISAD), School of Human Movement Science and Quality of Life, National University of Costa Rica, Heredia 86-3000, Costa Rica; drojasv@una.cr; 4 Updates for Sport Training and Physical Conditioning Research Group (GAEDAF), Sport Science Faculty, University of Extremadura, 10005 Caceres, Spain; 5 Optimization of Training and Sport Performance Research Group (GOERD), Department of Didactics of Plastic, Music and Body Expression, Sport Science Faculty University of Extremadura, 10005 Caceres, Spain

**Keywords:** competition, contextual variables, non-lineal locomotion, inertial devices, monitoring, sports performance

## Abstract

The study of the contextual variables that affect soccer performance is important to be able to reproduce the competition context during the training sessions. Therefore, the aim of the present study was to evaluate the effect of match outcome as related to goal difference (large win, >2 goals, LW; narrow win, 1–2 goals, NW; drawing, D; narrow loss, 1–2 goals, NL; or large loss, >2 goals, LL), match location (home, H; away, A; neutral, N), type of competition (international, INT; national, NAT; friendly, F), phase of the season (summer preseason, SPS; in-season 1, IS1; winter preseason, WPS; in-season 2), and the field surface (natural grass, NG; artificial turf, TF) on the change of direction (COD) and centripetal force (CentF) generated during official games. Thirty male elite-level soccer players (age: 26.57 ± 5.56 years) were assessed while using WIMU PRO^TM^ inertial devices (RealTrack Systems, Almeria, Spain) in 38 matches during the 2017–2018 season, selecting for analysis the number of COD at different intensities and the CentF, depending on the turn direction. Statistical analyses comprised a one-way ANOVA with the Bonferroni post-hoc and *t*-test for independent samples. The main results showed that the match outcome (*ω_p_*^2^ = 0.01–0.04; NW = D = NL > LL), match location (*ω_p_*^2^ = 0.01–0.06; A = N > H), type of competition (*ω_p_*^2^ = 0.01–0.02; INT > NAT > F), and period of the season (*ω_p_*^2^ = 0.01–0.02; SPS = IS1 = WPS > IS2) all exert some influence. No effect was found for the playing surface. Therefore, match outcome, match location, type of competition, and period of the season influence the demands of centripetal force and changes of direction. These aspects should be considered in the design of training sessions and microcycle workload planning during the season to improve competitive success.

## 1. Introduction

Outdoor team sports, especially soccer, are characterized by the performance of high-intensity intermittent efforts, where repeated sprints, rapid accelerations and decelerations, rapid changes of directions, jumps, throws, and duels are continuously performed with incomplete recovery [1]. These abilities and locomotion are dynamic and unpredictable in addition to varying in the intensity and duration during the competition [2].

Among these, the ability to perform a sprint and change direction is considered to be essential for physical performance in soccer, allowing for the players to get ahead in the duel over the ball and create or block clear opportunities to achieve a goal [3,4]. This ability requires a high mechanical load, such as eccentric contractions and a physiological load [5], which are reflected in an increase in muscle damage during training sessions [6] and official matches [7].

Research into external workload during match play has tended to focus on distance covered, speed, or number of efforts above a predetermined threshold [8,9]. Furthermore, the approach that many authors have used in these studies often focuses on linear actions. However, linear actions are trivial and, due to the open nature of soccer, the critical actions will often be curvilinear [10]. In this respect, it has been shown that soccer players perform hundreds of changes of direction throughout the game [2]. Of note, approximately 85% of the actions that are executed at maximum velocity in elite teams consist of curvilinear sprints, that is to say the upright running portion of the sprint completed with the presence of some degree of curvature [11]. Nonetheless, there is a lack of research regarding the performance of changes of direction and centripetal force generated in official competition despite their critical importance. 

On the other hand, curvilinear displacement technique presents different kinetic and kinematic features [12,13,14]. These differences can be reviewed in the work of Churchil et al., (2015) [15], but a key factor is the trunk rotation and, consequently, the neuromechanical requirements that this implies. For this reason, linear and nonlinear sprint performances embody different physical and technical capabilities, and they should be independently assessed and trained. A proper design and management of training loads are important to keep the player available the maximum time possible, especially in elite soccer [16]. For this, it is recommended to monitor demands while using electronic devices [17,18], which are portable and non-invasive, and they contain different sensors (accelerometers, gyroscopes, magnetometers, global positioning systems, and ultra-wide band sensors) for recording external workload (kinematic and neuromuscular) [17,19], and internal workload [20,21].

These internal and external workloads are influenced by different contextual variables, of which the most relevant are: (a) loss of performance throughout the game, with a decrease between the first and second half in variables, such as distance traveled, high intensity activity, or sprints [22,23]; (b) the specific demands depending on the game position in relation to playing formation and competitive dynamics [23,24,25]; (c) the period of the season, higher demands being found at the end of the season [26]; (d) match location, higher demands being found for home than away matches [27]; (e) game outcome: wins are less exhausting than draws or defeats [28,29,30]; (f) the competitive level [31]; or, (g) the playing surface, with greater demands as the playing surface is softer and unstable (sand > natural grass > artificial turf > hard surface) [32].

It is hypothesized that they can also have an effect on the performance of non-linear locomotion because the influence of these variables has already been studied previously as having an effect on other physiological and kinematic parameters in soccer. Therefore, the objective of the present study was to analyze the effect of the contextual variables of match outcome, match location, type of competition, period of the season and playing surface on the centripetal force, and changes of direction recorded with electronic devices during a competitive season in elite soccer.

## 2. Materials and Methods

### 2.1. Participants

Thirty elite-level soccer players participated voluntarily in this research (age: 26.57 ± 5.56 years; height: 1.82 ± 0.05 meters; body mass: 77.2 ± 2.76 kg.; fat percentage: 8.44 ± 1.08%; body mass index: 22.31 ± 1.32 kg/m^2^). All of the players belonged to the same team, which participated in the Russian Premier League, Russian Cup, Champions League and Europa League in the 2017–2018 season. All of the participants had to meet the requirement of the absence of any type of physical limitations or musculoskeletal injuries that could affect the workload monitoring during official matches. Besides, to be included in the final analysis, players had had to play the full length of a game in order to be included in the analysis (~90 min.). The Bioethics Commission of the University approved the study, which was conducted according to the Declaration of Helsinki (Reg. Code 2595-2019). The participants were informed of the risks and discomforts that are associated with testing and provided written informed consent.

### 2.2. Material

#### 2.2.1. Anthropometric Measurements 

Height was measured to the nearest 0.5 cm during a maximal inhalation while using a wall-mounted stadiometer (SECA, Hamburg, Germany). Body mass was obtained using an eight-electrode segmental body composition monitor BC-601 model (TANITA, Tokyo, Japan).

#### 2.2.2. Inertial Device 

Each participant wore an inertial device, called a WIMU PRO^TM^ (RealTrack Systems, Almeria, Spain), which was placed on the upper back (interscapular line, T2-T4 vertebrae) in a specific harness to achieve the best GPS signal reception [33]. This device has been given the FIFA certificate for use during official competition and it is composed of different sensors: (a) four triaxial accelerometers (1000 Hz) with a full-scale output range of ±16, ±16, ±32, and ±400 g; (b) three triaxial gyroscopes (1000 Hz) with a full-scale output range of 2000 degrees/seconds; (c) a three-dimensional (3D) magnetometer; (d) a 10 Hz GPS chip; and, (e) a 20 Hz UWB chip. During the present study, the sampling frequency of the microelectromechanical sensors (accelerometer, gyroscope, and magnetometer) was 100 Hz, and the GPS sensor was 10 Hz.

The accuracy and reliability of the GPS, gyroscope and accelerometer in the inertial device have been previously evaluated with satisfactory results [34,35,36]. Besides, the inter-unit reliability (ICC = 0.75–0.96) and the validity (Bias, counter-clockwise = −2.19N; clockwise = 1.75N) for measuring centripetal force have been analyzed with satisfactory results (Unpublished data). Two matches were deleted from the final analysis, because the operating conditions for the GPS were not optimal [37]. During the recording process, the GPS sensor was connected to 12.8 ± 2.5 satellites and the horizontal geometric dilution of precision (HGDOP) for Global Navigation Satellite Systems (GNSS) was practically ideal, with a result of 0.96 ± 0.14 [38].

Prior to placement, the inertial devices were calibrated and then synchronized following manufacturer guidelines. For this, three aspects were considered to improve the data accuracy: a) to leave the device immobile for 30 s; (b) on a flat surface; and, (c) without electromagnetic devices nearby [38,39]. 

### 2.3. Variables

#### 2.3.1. Non-Linear Locomotion

Table 1 shows the variables selected to analyze non-linear locomotion. All of them were calculated through two main variables: (a) *Centripetal force (CentF)*, as the force or force component acting on a moving object on a curvilinear trajectory which is directed towards the center of the curvature of the path [40]; and, (b) *Change of direction (COD)*, considered as the specific event where the athlete performed “a movement or skill to change direction, velocity, and locomotion mode” [41]. 

#### 2.3.2. Contextual Variables

In the present study, the influence of the following contextual variables related with the official competition dynamics was analyzed:

- *Period of the season*. This variable is divided into four periods due to the climatic conditions that do not allow playing matches in the winter period: (a) Summer preseason, SPS (July–August) (*n* = 5); (b) In-season 1, IN1 (September to December) (*n* = 11); (c) Winter preseason, WPS (January–February) (*n* = 9); and, (d) In-season 2, IN2 (March to June) (*n* = 13).
- *Type of competition*. With respect to the nature of the competition, it is divided into three groups: (a) international, INT (Champions League and Europa League) (*n*=7); (b) national, NAT (League and Cup) (*n* = 15); and, (c) friendly matches, F (*n* = 16).
- *Match location*. Classified into three groups: (a) home, H (*n* = 7); (b) away, V (*n* = 17); and, (c) neutral, N (*n* = 14).
- *Match outcome*. As a function of the goal difference in the final result of the game, this variable was divided into five groups: (a) large win, LW, winning the game with a difference of over two goals (*n* = 10); (b) narrow win, NW, winning the game with a difference of between one and two goals (*n* = 12); (c) drawing, D, the same number of goals by each team or no goals in the game (*n* = 10); (d) narrow loss, NL, losing the game with a difference of between 1 and 2 goals (*n* = 6); (e) large loss, LL, losing the game with a difference of over 2 goals (*n* = 0).
- *Playing surface*. Divided according to the different types of surface allowed by the Federation International of Football Associations (FIFA) into two types: (a) natural grass, NG (*n* = 31), and (b) artificial turf, TF (*n* = 7).

### 2.4. Procedures

The data were collected during the 2017–2018 season in the matches played by a male elite-level soccer team that participated in domestic and international competitions. All of the matches were played on natural grass or artificial turf according to FIFA quality standards. The playing formation used during the investigation was 4-4-2. The players were familiar with the functions of their specific position and the playing formation.

The players arrived in the locker room 45 minutes before the start of the matches for placement of the inertial devices. The devices were worn during the warm-up and the entire match. The information from the devices was downloaded to a computer at the end of the match. All of the raw files were synchronized with the time selection of first and second periods that were made in real-time while using a tablet with SVIVO^TM^ software (RealTrack Systems, Almeria, Spain). Time selections and raw files were synchronized in the SPRO^TM^ software (RealTrack Systems, Almeria, Spain). Subsequently, the data were exported and entered into an Excel database. Finally, the database was imported into the SPSS software (IBM Corporation, Armonk, USA) for statistical analysis. 

### 2.5. Data Analysis

The descriptive analysis was performed and is presented as mean (M) ± standard deviation (SD) for each variable. Data distribution was subsequently analyzed by the Kolmogorov–Smirnov test and data homoscedasticity by the Levene test to confirm a normal distribution. A one-way ANOVA was performed with the Bonferroni post-hoc for the comparative analysis among contextual variables (match outcome, match location, type of competition, and period of the season) and non-linear locomotion (change of direction and centripetal force), while an independent sample *t*-test was used for the playing surface analysis. The magnitude of differences was obtained while using the statistical test partial omega squared (ω_p_^2^) interpreted as: >0.01 low; >0.06 moderate; or >0.14 high; and, Cohen’s d (*d*) interpreted as: trivial (0–0.19), low (0.20–0.49), moderate (0.50–0.79), or high (>0.80) [42]. The statistical analyses were performed with IBM SPSS Statistics software (release 24.0; SPSS Inc., Armonk, NY, EE. UU.). The statistical significance was established at *p* < 0.05 and the *p* values were corrected for multiple comparisons by the software.

## 3. Results

### 3.1. Period of the Season

Table 2 shows the comparative analysis between the non-linear locomotion (change of direction and centripetal force) and the period of the season (summer preseason, in-season 1, winter preseason, in-season 2). The highest demands were found in the winter preseason, showing significant differences in +CentF_AVG_, COD_SPRINT_, R_60_COD_HIA_, R_60_COD_SPRINT_ (*F >* 3.59; *ω_p_*^2^ = 0.01–0.02, *low effect*) with respect to the in-season 2. However, similar demands were found between in-season 1 and in-season 2, except in +CentF_AVG_ and R_20_COD_HIA_, with greater values in the first part of the season (*F >* 2.89; *ω_p_*^2^ = 0.01, *low effect*). 

### 3.2. Match Location

Table 3 shows the comparative analysis between centripetal force and change of direction in relation to match location (home, away, neutral). The home matches recorded lower demands with statistically significant differences with respect to away and neutral conditions in all non-linear performance variables (*F >* 3.02; *ω_p_*^2^ = 0.01–0.06, *moderate to low effect*), except in Difference (+% vs. −%), COD, R_20_COD, and R_60_COD. The workload demands in matches played away and neutral conditions were similar and no differences were found between both conditions. 

### 3.3. Match Outcome

Table 4 shows the comparative analysis between non-linear locomotion (centripetal force and change of direction) as a function of match outcome (drawing, narrow win, large win, narrow loss, and large loss). The effect of match outcome produced differences in +CentF_MAX_, −CentF_MAX_, +CentF_AVG_, −CentF_AVG_, Difference (+% vs. −%), R_60_COD_HIA_, and R_60_COD_SPRINT_ with a lower effect size (*F =* 2.95–10.77; *ω_p_*^2^ = 0.01–0.04). Specifically, large wins presented the lowest demands, obtaining statistically significant differences in +CentF_AVG_ and −CentF_AVG_ with drawing, narrow wins and narrow losses, in −CentF_MAX_ with narrow wins and losses, in +CentF_MAX_ and R_60_COD_SPRINT_ with drawing, and in R_60_COD_HIA_ with narrow losses.

### 3.4. Type of Competition

Table 5 shows the results of the comparative analysis between the centripetal force and change of direction in relation to type of competition (international, national, and friendly matches). In general, the greatest demands were experienced in international matches. Specifically, significant differences were found in Difference (+% vs. −%), R_60_COD_HIA_, and R_20_COD_SPRINT_ with higher values in international matches with respect to national and friendly matches with a low effect size (*F >* 2.95; *ω_p_*^2^ = 0.01–0.02).

### 3.5. Playing Surface

Table 6 shows the results of the comparative analysis between non-linear locomotion (centripetal force and change of direction) as a function of playing surface (natural grass and artificial turf). No differences were found in the non-linear workload between both types of surfaces (*t <* 2.77; *d* *<* 0.14).

## 4. Discussion

Soccer performance consists of a combination of physical characteristics, technical skills, and tactical organization [26,43]. Regarding the tactical organization and technical skills, recent studies have indicated that greater technical efficiency [31,43] and better synchronization among team players [44,45] are associated with a greater probability of achieving success. Related with physical characteristics, previous research has determined that the winning teams perform better in high intensity locomotion (above 16 km/h) and speed changes (accelerations and decelerations above 3 m/s) [29,46]. Conversely, a greater volume of locomotion (distance, accelerations, decelerations) is associated with a poorer performance due to the losing teams attempt to recover from an unfavorable position, both in small-sided games [47,48] and during official matches [49,50]. 

Regarding physical performance indicators, centripetal force and changes of direction are two essential components in team sports, allowing for the players to get ahead in the duel over the ball and create or block clear opportunities to get a goal [3,4]. For this reason, different research has investigated to what extent the training of different variables influences the performance of changes of direction, such as strength training of the lower limbs [51], trunk stability [52], or gait biomechanics [12,13,14]. However, the question is whether it is worth improving performance in changes of direction and centripetal force, and if they have an effect on performance during the competition. Therefore, the purpose of this study was to evaluate the effect of contextual variables, such as the period of the season, match outcome, match location, type of competition, and type of surface in non-linear locomotion in an elite-level soccer team. All of the contextual variables had an effect on non-linear locomotion, except playing surface. The greatest demands were experienced in the winter preseason (Europa League final phase and friendly matches against elite-level international teams between January and February) with drawing or narrow wins and losses, in international matches and away or in neutral conditions.

A significant increase of centripetal force and COD values were reported during in-season 1 and the winter preseason with respect to the summer preseason and in-season 2. This could confirm two findings that were developed by previous studies: (i) on the one hand, some authors suggest that the locomotion requirements in matches or the team’s level could be related with the activity of the opposing team [53], (ii) on the other hand, Rampinini et al. [26] show how elite-level teams achieved higher values in physical performance variables (total distance, high-intensity distance, sprinting distance) when they faced better quality teams. Therefore, these results are in the same line as these studies, which show how the opponent influences physical development in the competition, when the obvious thing would have been to increase throughout the season. However, small effect size differences in non-linear locomotion suggest that the influence of the period of the season on general fatigue or the required work rate is relatively small. Even so, the current results indicate that elite-level soccer players must be physically prepared, so that they can cover not only greater distances and at greater intensity, but also develop a more non-linear locomotion with changes of direction.

Similarly, Rampinini et al. [26] showed that the type of competition is related to an opposing team with a higher or lower quality, both at the physical and technical-tactical level. This has been found in the results of the present study, where the highest demands in centripetal force and changes of direction were found in international matches, then in national matches and, finally, with the lowest demands, in friendly matches. It is suggested that the training microcycles that end with an international match require a specific design with higher demands in all types of locomotion by the soccer player and at all intensities, specifically in non-linear trajectories, as shown in this study. 

With respect to the match location, from the study by Schwartz and Barsky [54], the advantage in the home condition has been identified from amateur to professional level in most sports [55]. Specifically for soccer, it has been shown that this home advantage has existed since the beginning of the soccer league in England in 1888–1889 and has since continued at all levels of professional matches [56]. Even with this evidence, there is a lack of research on this phenomenon, and only a few studies have addressed its influence on external workload in soccer players. The present results showed a significant decrease in the performance of non-linear trajectories in official matches in the home condition (*p* < 0.05). In contrast, previous studies found higher values in workload demands, such as maximum speed and percentage of high intensity actions (*p* < 0.05) [50,57], and technical-tactical indicators, such as on-goal shooting and goal-scoring opportunities [58]. Although the present findings require a more detailed study (only one elite-level team during a season), they are suggesting in a low to moderate magnitude (*ω_p_*^2^ = 0.01 to 0.06) that non-linear locomotion demands do not follow the same dynamics as linear locomotion. This phenomenon also occurred with the match outcome. While in the study by Aquino et al. [50] higher values were reported in high intensity variables and total distance covered, in the present study lower values were found when the team was winning, especially in large wins (a difference of over two goals).

Finally, in the type of surface analysis, although higher values were reported in centripetal force and changes of direction when the match was played on artificial turf with respect to natural grass, the null hypothesis that a different surface was going to influence the centripetal force and the changes of direction performed during official games was rejected. This comparison of surfaces has been investigated in other approaches, such as: (a) the incidence of injury with no differences between surface, but a higher number of instances of low back and chronic pain was found on artificial turf [59]; (b) speed performance, with faster values on artificial turf so that greater skill is needed in order to avoid injuries [60]; and (c) fatigue with greater acute decrement in hamstring peak torque on natural grass, but no delay in the recovery process was found between surfaces. Therefore, although there exist differences in fatigue, injury incidence, and linear locomotion, during official matches the non-linear locomotion demands are similar and the training process should address these findings to achieve an optimum performance and decrease the injury risk, depending on the type of surface. 

After the analysis of the competition, the coach will be able to know the specific profile of his players in non-linear locomotion, both in the changes of direction made and in the centripetal force generated [61]. While using this information, it would be advisable to design tasks either in specific game situations (small-sided games and specific individual and collective tasks with the ball) or in others (curvilinear locomotion at maximum intensity, zigzag movements).

While the results of this study have provided information about the non-linear performance of professional players that belonged to an elite-level soccer team, thanks to the use of electronic performance and tracking systems, and while considering multiple contextual factors, such as period of the season, type of competition, match location, match outcome, and playing surface, some limitations to the study must be acknowledged. One of the limitations in this research concerns the sample studied. Only the non-linear performance of one soccer team through centripetal force and change of direction has been analysed during an entire season because of limited access to elite-level soccer players. This meant that only one playing system and one playing style of the team conditioned the results of the study. Despite this fact, the authors did not influence the natural dynamics of the competition, giving an ecological treatment to the study. Finally, data collection was performed following the same protocol throughout the matches, but the environmental influence was not controlled.

## 5. Conclusions

The contextual variables analyzed: period of the season, match outcome, match location, and type of competition had an effect on non-linear locomotion, such as changes of direction and the centripetal force generated during the competition. In contrast, the playing surface had no influence on these demands. Therefore, the following suggestions could be considered for using the information obtained in the present study in a practical way for performance improvement and proper training planning based on the conclusions obtained:

- The period of the season had a significant effect on the non-linear locomotion workload. A progressive increase in change of direction and centripetal force performance was found in the team studied from summer preseason to winter preseason, maintaining these values until the end of the season. Following the results that were obtained, a progressive increase in non-linear locomotion workload, reaching the highest values in winter preseason, allows for maintaining performance between in-season periods.
- Match location had a direct effect on workload management in every microcycle, with the need to increase the load of these individual technical abilities when the end of the competitive microcycle coincides with an official away or neutral location match.
- The non-linear locomotion performance determines the soccer match outcome. A large goal difference both in the winning and losing team produced a drastic reduction in the centripetal force and a change of direction demands. In this respect, it is interesting to design game-based tasks and conditional tasks that represent different match outcome scenarios to prepare the players both physically and psychologically for these contexts during the competition with the aim of maintaining the best competitive performance.
- Regarding the type of competition, international matches required higher demands in comparison to national and friendly matches. In this respect, a higher-level competition needs a special preparation period with an increase of non-lineal locomotion demands with the aim of facing the match in optimal conditions.
- The type of surface did not show differences in the performance of non-linear locomotion. Therefore, due to this peculiarity that occurs in countries with cold climates, where low temperatures complicate the maintenance of natural grass, combined training on artificial and natural surfaces is necessary to adapt the player to both, because they present the same demands for changes of direction and centripetal force.

## Figures and Tables

**Table 1:** Description of centripetal force and change of direction analyzed variables during the present research.

Variable	Sub-Variable	Description
Centripetal Force (CentF)	+CentF_AVG_	Average of the centripetal force generated by the player throughout the game when he turned clockwise.
	-CentF_AVG_	Average of the centripetal force generated by the player throughout the game when he turned counterclockwise.
	+CentF_MAX_	Maximum centripetal force generated by the player throughout the game when he turned clockwise.
	-CentF_MAX_	Maximum centripetal force generated by the player throughout the game when he turned counterclockwise.
	DifCentF_AVG_	Average difference of centripetal force as a function of the direction of rotation.
Changes of Direction (CODs)	CountCOD	Number of total changes of direction performed in a match.
	CountCOD_HIA_	Number of total changes of direction performed in a match at high intensity (above 16 km/h).
	CountCOD_SPRINT_	Number of total changes of direction performed in a match at maximum intensity (above 21 km/h).
	R_20_COD	Number of total changes of direction performed in a match with a recovery time less than 20 s.
	R_60_COD	Number of total changes of direction performed in a match with a recovery time less than 60 s.
	R_20_COD_HIA_	Number of total changes of direction performed in a match at high intensity (above 16 km/h) with a recovery time less than 20 s.
	R_60_COD_HIA_	Number of total changes of direction performed in a match at high intensity (above 16 km/h) with a recovery time less than 60 s.
	R_20_COD_SPRINT_	Number of total changes of direction performed in a match at maximum intensity (above 21 km/h) with a recovery time less than 20 s.
	R_60_COD_SPRINT_	Number of total changes of direction performed in a match at maximum intensity (above 21 km/h) with a recovery time less than 60 s.

**Table 2:** Descriptive and comparative analysis of centripetal force and changes of direction related to the period of the season.

Variables	Summer Preseason (*n* = 42) M ± DE	In-Season 1 (*n* = 86) M ± DE	Winter Preseason (*n* = 60) M ± DE	In-Season 2 (*n* = 117) M ± DE	*F*	*p*	*ω_p_* ^2^
+CentF_MAX_	937.14 ± 362.75	949.43 ± 285.89	**958.75 ± 350.13**	922.14 ± 274.43	1.76	0.151	0.00
−CentF_MAX_	−925.62 ± 281.00	**−932.59 ± 267.19**	−926.77 ± 314.83	−899.68 ± 264.68	1.66	0.171	0.00
+CentF_AVG_ *	209.30 ± 19.19	**210.48 ± 19.20** ^d^	210.14 ± 17.71 ^d^	207.28 ± 17.41	4.16	0.006	0.02
−CentF_AVG_	−208.15 ± 19.94	**−209.75 ± 17.56**	−209.61 ± 24.11	−207.60 ± 15.95	1.73	0.159	0.00
Difference (+% vs. −%) *	1.46 ± 12.42	−0.42 ± 12.82	0.52 ± 17.45	**2.12 ± 10.32** ^b^	3.68	0.012	0.01
COD	592.52 ± 153.45	622.96 ± 161.13	**657.00 ± 165.70**	637.08 ± 159.79	0.69	0.557	0.00
COD_HIA_ (>16 km/h)	57.84 ± 15.85	62.24 ± 17.11	**62.96 ± 16.54**	55.64 ± 16.08	1.66	0.173	0.00
COD_SPRINT_ (>21 km/h) *	14.4 ± 4.74	15.44 ± 5.08	**18.56 ± 6.03** ^d^	14.96 ± 5.44	3.85	0.009	0.01
R_20_COD	528.20 ± 138.21	559.80 ± 146.27	550.84 ± 138.38	**572.48 ± 144.67**	0.35	0.793	0.00
R_60_COD	56.52 ± 15.12	56.88 ± 15.85	53.72 ± 14.41	**59.00 ± 15.30**	0.81	0.488	0.00
R_20_COD_HIA_ *	16.56 ± 5.16	**19.68 ± 6.22** ^d^	18.92 ± 5.64	16.36 ± 5.70	2.89	0.034	0.01
R_60_COD_HIA_ *	11.36 ± 3.61	11.80 ± 4.07	**13.20 ± 4.18** ^d^	10.28 ± 3.71	3.59	0.013	0.01
R_20_COD_SPRINT_ *	2.44 ± 1.11	2.84 ± 1.37	**4.04 ± 1.78** ^a,b^	3.12 ± 1.66	5.59	0.001	0.02
R_60_COD_SPRINT_ *	0.80 ± 0.60	1.04 ± 0.69	**1.44 ± 0.84** ^a,d^	0.88 ± 0.60	5.26	0.001	0.02

**Note.** M: Mean; SD: Standard deviation; CentF: Centripetal force (+: clockwise, −: counterclockwise); COD: Changes of direction; RCOD: Ability to repeat a change of direction; *p*: *p* value; F: ANOVA’s F value; ω_p_^2^: Partial omega squared; * Statistical differences (*p* < 0.05) with ^a^ Summer preseason (july-august); ^b^ In-season 1 (September-December), ^c^ Winter preseason January-February), ^d^ In-season 2 (March-June); Bold numbers represent maximum values of each variable.

**Table 3:** Descriptive and comparative analysis of centripetal force and changes of direction related to the match location.

Variables	Home (*n* = 77) M ± DE	Away (*n* = 138) M ± DE	Neutral (*n* = 90) M ± DE	*F*	*p*	*ω_p_* ^2^
+ CentF_MAX_ *	902.71 ± 310.53	952.41 ± 269.57 ^a^	**955.54 ± 358.17** ^a^	4.69	0.009	0.02
−CentF_MAX_ *	−893.29 ± 276.00	−920.52 ± 257.57	**−934.13 ± 318.01**	3.02	0.049	0.01
+ CentF_AVG_ *	205.07 ± 18.35	**210.51 ± 17.92** ^a^	210.20 ± 18.20 ^a^	15.13	<0.001	0.05
−CentF_AVG_ *	−206.20 ± 15.78	−209.36 ± 17.05 ^a^	**−209.88 ± 24.30**	5.25	0.005	0.02
Difference (+% vs. −%)	1.13 ± 10.90	**1.25 ± 13.25**	0.09 ± 16.33	1.81	0.164	0.00
COD	601.60 ± 148.26	644.40 ± 165.69	**645.60 ± 163.79**	0.79	0.453	0.00
COD_HIA_ (>16 km/h) *	51.84 ± 14.68	**62.80 ± 17.45** ^a^	61.32 ± 16.17 ^a^	4.32	0.013	0.02
COD_SPRINT_ (>21 km/h) *	11.32 ± 3.69	**17.44 ± 5.89** ^a^	17.24 ± 5.67 ^a^	13.12	<0.001	0.04
R_20_COD	540.28 ± 134.50	**579.60 ± 150.41**	537.84 ± 135.86	1.35	0.260	0.00
R_60_COD	56.16 ± 14.54	**58.56 ± 15.95**	53.84 ± 14.46	1.24	0.290	0.00
R_20_COD_HIA_ *	14.80 ± 5.10	**19.72 ± 6.30** ^a^	18.08 ± 5.42 ^a^	6.78	0.001	0.02
R_60_COD_HIA_ *	9.88 ± 3.64	11.76 ± 4.05	**12.84 ± 4.01** ^a^	4.57	0.010	0.02
R_20_COD_SPRINT_ *	1.60 ± 0.78	**3.80 ± 1.77** ^a^	3.52 ± 1.61 ^a^	19.31	<0.001	0.06
R_60_COD_SPRINT_ *	0.64 ± 0.45	1.16 ± 0.71 ^a^	**1.32 ± 0.82** ^a^	8.14	<0.001	0.03

**Note.** M: Mean; SD: Standard deviation; CentF: Centripetal force (+: clockwise, −: counterclockwise); COD: Changes of direction; RCOD: Ability to repeat a change of direction; *p*: *p* value; F: ANOVA’s F value; ω_p_^2^: Partial omega squared; * Statistical differences (*p* < 0.05) with ^a^ Home, ^b^ Away, ^c^ Neutral; Bold numbers represent maximum values of each variable.

**Table 4:** Descriptive and comparative analysis of centripetal force and changes of direction related to the match outcome.

Variables	Drawing (*n* = 76) M ± DE	Narrow Win (*n* = 104) M ± DE	Large Win (*n* = 77) M ± DE	Narrow Loss (*n* = 48) M ± DE	*F*	*p*	*ω_p_* ^2^
+ CentF_MAX_ *	**959.73 ± 306.04** ^c^	952.25 ± 349.73	912.77 ± 278.64	959.67 ± 290.31	2.95	0.032	0.01
−CentF_MAX_ *	−903.03 ± 250.49	**−950.78 ± 328.78** ^a,c^	−883.64 ± 259.06	−949.03 ± 257.87 ^c^	8.45	<0.001	0.03
+ CentF_AVG_ *	**211.71 ± 17.82** ^c^	209.84 ± 19.09 ^c^	206.07 ± 16.35	210.46 ± 19.20 ^c^	10.77	<0.001	0.04
−CentF_AVG_ *	−209.44 ± 17.22 ^c^	**−211.18 ± 24.52** ^c^	−205.37 ± 15.77	−209.00 ± 17.41 ^c^	10.46	<0.001	0.04
Difference (+% vs. −%) *	1.57 ± 12.29 ^b^	−0.99 ± 15.67	**2.53 ± 13.61** ^b^	0.76 ± 13.85	8.48	<0.001	0.03
COD	627.96 ± 157.65	628.40 ± 159.10	**654.80 ± 167.42**	647.40 ± 168.64	0.27	0.847	0.00
COD_HIA_ (>16 km/h)	63.16 ± 16.79	58.76 ± 16.29	56.00 ± 15.58	**65.48 ± 18.31**	1.69	0.167	0.00
COD_SPRINT_ (>21 km/h)	**17.76 ± 5.87**	16.04 ± 5.44	14.40 ± 5.10	16.16 ± 5.32	2.03	0.108	0.00
R_20_COD	544.16 ± 137.29	561.04 ± 143.48	556.32 ± 142.00	**580.72 ± 152.70**	0.25	0.860	0.00
R_60_COD	58.64 ± 15.23	53.36 ± 14.37	57.88 ± 15.31	**59.88 ± 17.37**	1.32	0.267	0.00
R_20_COD_HIA_	18.84 ± 5.81	18.00 ± 5.76	16.32 ± 5.31	**20.92 ± 6.77**	2.42	0.064	0.00
R_60_COD_HIA_ *	12.60 ± 4.06	11.12 ± 3.79	10.52 ± 3.69	**14.00 ± 4.70** ^c^	3.70	0.011	0.01
R_20_COD_SPRINT_	**3.44 ± 1.68**	3.41 ± 1.63	2.92 ± 1.44	3.01 ± 1.40	1.01	0.386	0.00
R_60_COD_SPRINT_ *	**1.24 ± 0.80** ^c^	1.24 ± 0.73 ^c^	0.68 ± 0.49	1.16 ± 0.78	4.86	0.002	0.02

**Note.** M: Mean; SD: Standard deviation; CentF: Centripetal force (+: clockwise, −: counterclockwise); COD: Changes of direction; RCOD: Ability to repeat a change of direction; *p*: *p* value; F: ANOVA’s F value; ω_p_^2^: Partial omega squared; * Statistical differences (*p* < 0.05) with ^a^ Drawing, ^b^ Narrow win, ^c^ Large win and ^d^ Narrow loss; Bold numbers represent maximum values of each variable.

**Table 5:** Descriptive and comparative analysis of centripetal force and changes of direction related to the type of competition.

Variables	International (*n* = 77) M ± SD	National (*n* = 138) M ± SD	Friendly (*n* = 90) M ± SD	*F*	*p*	*ω_p_* ^2^
+ CentF_MAX_	935.96 ± 203.33	**947.15 ± 285.86**	943.61 ± 335.64	0.56	0.945	0.00
- CentF_MAX_	−910.95 ± 239.23	**−924.37 ± 272.68**	−919.50 ± 296.62	0.12	0.892	0.00
+ CentF_AVG_	**212.48 ± 15.96**	209.60 ± 18.83	209.17 ± 17.91	0.97	0.380	0.00
- CentF_AVG_	−208.93 ± 14.48	**−209.28 ± 17.33**	−208.83 ± 21.82	0.14	0.872	0.00
Difference (+% vs. −%) *	**4.88 ± 10.34** ^b,c^	0.27 ± 12.34	0.90 ± 15.33	2.95	0.050	0.01
COD	**709.28 ± 186.17**	633.08 ± 163.04	636.88 ± 160.31	0.37	0.695	0.00
COD_HIA_ (>16 km/h)	**75.00 ± 21.57**	59.24 ± 16.75	60.06 ± 16.08	1.50	0.224	0.00
COD_SPRINT_ (>21 km/h)	**21.56 ± 7.54**	15.96 ± 5.54	16.02 ± 16.08	1.78	0.168	0.00
R_20_COD	**644.28 ± 170.51**	567.80 ± 147.44	546.81 ± 5.30	1.00	0.370	0.00
R_60_COD	**59.56 ± 15.73**	59.04 ± 16.10	54.56 ± 137.60	1.44	0.238	0.00
R_20_COD_HIA_	**24.16 ± 8.22**	18.36 ± 6.04	17.76 ± 14.46	2.05	0.129	0.00
R_60_COD_HIA_ *	**16.16 ± 5.40** ^b,c^	10.72 ± 3.79	12.24 ± 5.49	4.56	0.011	0.02
R_20_COD_SPRINT_ *	**5.72 ± 2.67** ^b,c^	3.28 ± 1.61	3.12 ± 4.02	4.64	0.010	0.02
R_60_COD_SPRINT_	**1.16 ± 0.78**	1.08 ± 0.69	1.12 ± 0.72	0.83	0.920	0.00

**Note.** M: Mean; SD: Standard deviation; CentF: Centripetal force (+: clockwise, −: counterclockwise); COD: Changes of direction; RCOD: Ability to repeat a change of direction; *p*: *p* value; F: ANOVA’s F value; ω_p_^2^: Partial omega squared; * Statistical differences (*p* < 0.05) with ^a^ International, ^b^ National, ^c^ Friendly; Bold numbers represent maximum values of each variable.

**Table 6:** Descriptive and comparative analysis of centripetal force and changes of direction related to playing surface.

Variables	Natural Grass (*n* = 77) M ± SD	Artificial Turf (*n* = 138) M ± SD	*t*	*p*	*d*
+ CentF_MAX_	944.59 ± 325.44	**945.28 ± 272.29**	−0.04	0.966	−0.00
- CentF_MAX_	**−923.53 ± 296.25**	−911.16 ± 244.47	−0.84	0.401	−0.05
+ CentF_AVG_	209.27 ± 18.62	**209.95 ± 16.46**	−0.73	0.465	−0.04
- CentF_AVG_	−208.96 ± 21.02	**−209.17 ± 16.04**	0.25	0.834	0.01
Difference (+% vs. −%) *	0.38 ± 14.36	**2.32 ± 13.50**	−2.77	0.006	−0.14
COD	633.68 ± 161.54	**651.44 ± 163.52**	−0.54	0.596	−0.03
COD_HIA_ (>16 km/h)	58.92 ± 16.32	**64.12 ± 17.23**	−1.51	0.132	−0.08
COD_SPRINT_ (>21 km/h)	15.68 ± 5.37	**17.60 ± 5.81**	−1.67	0.095	−0.09
R_20_COD	549.36 ± 140.75	**588.40 ± 149.13**	−1.31	0.204	−0.07
R_60_COD	56.40 ± 15.27	**56.96 ± 14.95**	−0.17	0.860	−0.01
R_20_COD_HIA_	17.76 ± 5.77	**19.64 ± 5.95**	−1.55	0.128	−0.08
R_60_COD_HIA_	11.52 ± 3.91	**12.36 ± 4.14**	−0.98	0.340	−0.05
R_20_COD_SPRINT_	3.12 ± 1.56	**3.72 ± 1.63**	−1.83	0.067	−0.09
R_60_COD_SPRINT_	**1.12 ± 0.72**	1.08 ± 0.68	0.14	0.893	0.01

**Note.** M: Mean; SD: Standard deviation; CentF: Centripetal force (+: clockwise, −: counterclockwise); COD: Changes of direction; RCOD: Ability to repeat a change of direction; *p*: *p* value; t: independent samples *t*-test value; d: Cohen’s d effect size; * Statistical differences (*p* < 0.05) between groups; Bold numbers represent maximum values of each variable.
